# Supplementary material for: Novel Function of Nogo-A as Negative Regulator of Endothelial Progenitor Cell Angiogenic Activity: Impact in Oxygen-Induced Retinopathy
Source: Int J Mol Sci. 2023 Aug 24;24(17):13185. doi: 10.3390/ijms241713185 (PMC10488245; doi:10.3390/ijms241713185)
Supplement: Supplementary file 1 [file ijms-24-13185-s001.zip › ijms-2548941-supplementary.pdf]

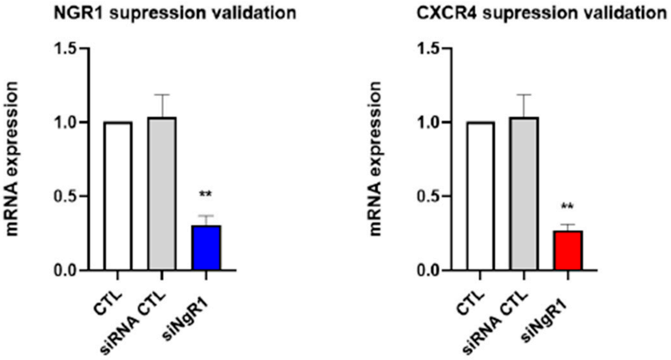

Supplemental Figure S1. Efficacy of siRNA-NgR1 and siRNA-CXCR4 in EPCs. qRT-PCR analyses of EPCs transfected with an siRNA-NGR1 or siRNA-CXCR4 24h after transfection.

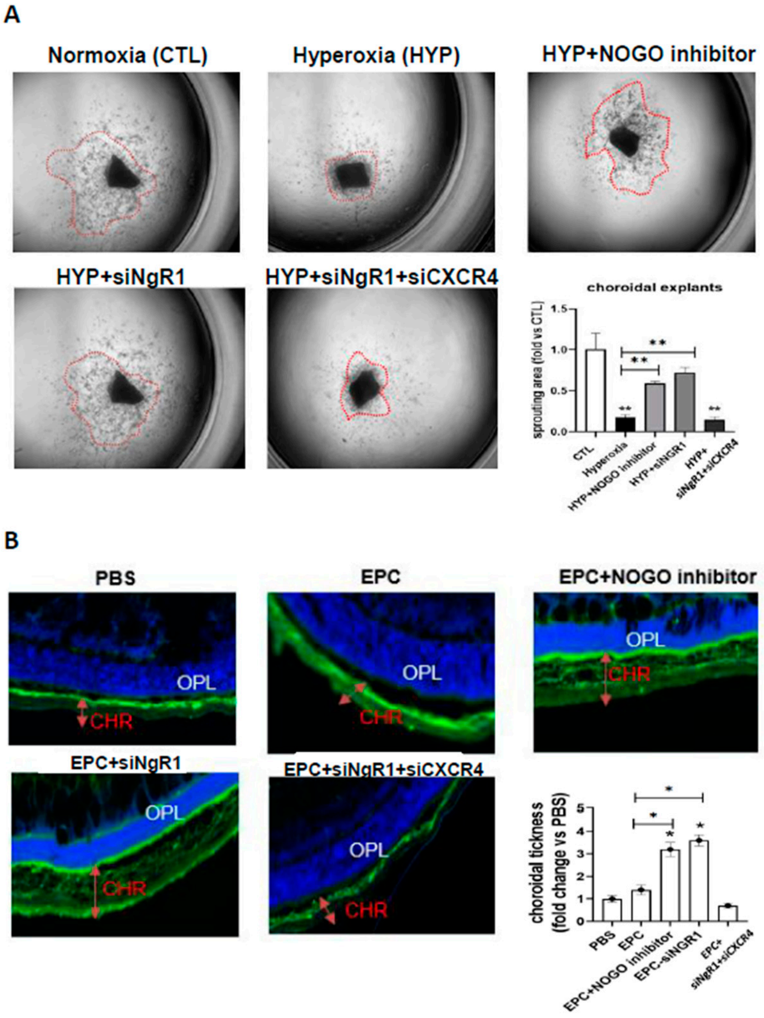

Supplemental Figure S2. Effects of NOGO/NgR1 inhibition on choroidal sprouting ex vivo (A) and on choroid thickness in rats subjected to OIR (B). A. Ex-vivo choroidal sprouting assay of P10-isolated choroid of normoxic rats; choroids were plated in Matrigel in complete EGM medium and treated with an NOGO inhibitor, siNgR1 or siNgR1+siCXCR4, and subjected to hyperoxia. B. Representative images and quantification analyses of choroidal thickness in retinal cross-section stained for vessel (isolectin positive, green) and DAPI in OIR-subjected rats treated with the same conditions. Data were mean ⊚ SEM. *$p<0.05$ or **$p<0.005$ vs PBS (control) or normoxic CTL. N=5 choroids.
